# Supplementary material for: On the mechanics of inhaled bronchial transmission of pathogenic microdroplets generated from the upper respiratory tract, with implications for downwind infection onset
Source: PLoS One. 2025 Nov 7;20(11):e0335962. doi: 10.1371/journal.pone.0335962 (PMC12594396; doi:10.1371/journal.pone.0335962)
Supplement: Attachment — Submitted filename: Rebuttal_Letter_Round2.pdf [file pone.0335962.s002.pdf]

SOUTH DAKOTA STATE UNIVERSITY

Saikat Basu, Ph.D.
Associate Professor
Mechanical Engineering
South Dakota State University
Crothers Hall 216, ME Box 2219
Brookings, South Dakota 57007, USA
P: 605-688-6868
Saikat.Basu@sdstate.edu

October 15, 2025

Subject: Rebuttal Letter (with Round 2 revision)

Dear Dr. Islam and the Reviewers,

I sincerely appreciate your inputs and suggested revisions for the manuscript titled "On the mechanics of inhaled bronchial transmission of pathogenic microdroplets generated from the upper respiratory tract, with implications for downwind infection onset". The revised manuscript (both marked-up and compiled versions) is enclosed herewith.

I have implemented all the suggested revisions pointed out in the 2nd round of peer review. The marked version of the manuscript has the track-change highlighted, as per the following color scheme:

- newly added material in 2nd round of revision, in green text
- deleted parts in 2nd round of revision, in magenta strike-through
- newly added references in 2nd round of revision, in green highlight

- newly added material in the 1st round of revision, in royal blue text
- deleted parts in the 1st round of revision, in red strike-through
- newly added references in the 1st round of revision, in royal blue highlight

Changes in the 2nd round of revision (comprising the latest revisions)

Point-by-point responses: Reviewers' comments (in black) + Author's responses (in green)

# Reviewer 1: Minor Revision

For any item you cannot fully address now, state it explicitly as a study limitation in the Discussion.
– I appreciate the reviewer's recommendation. All the limitations are now explicitly acknowledged and discussed in greater detail in the expanded Discussion section. In addition, as a summary, a new table (Table 3; see page 22 of the marked copy) has been included, featuring a domain-specific, itemized list of future research topics aimed at addressing the current limitations.

1. Insufficient experimental validation — Current text mentions only "representative" validation against a small published set; add quantitative comparisons (e.g., gamma-scintigraphy or 3D-printed cast tests) for penetration / deposition profiles. If not feasible, flag as a limitation.
– I thank the reviewer for the suggestion. The limitation concerning the representativeness of the experimental validation has now been emphasized in lines 550 – 554 (on page 19 in the marked-up copy). These lines are additions to the existing Discussion section on experimental validation. The rest of the paragraph (lines 554 – 571) discusses the prospect of gamma scintigraphy measurements in 3D-printed casts, with references to prior works.

2. No evaporation / thermo-hygroscopic growth modeling — The flow is isothermal and heat transfer is discounted, so droplet size evolution is ignored; add a coupled heat/mass-transfer model or at least a sensitivity analysis to

RH/temperature. If not feasible, note as a limitation.
– This is an excellent recommendation. Accordingly, a new paragraph has been added to the Discussion (see lines 639 – 649, on page 21 in the marked copy). The paragraph explicitly discusses the limitation around the lack of evaporation / thermo-hygroscopic modeling. For future work, it also comments on a coupled heat and mass transfer model and on sensitivity analysis with parameters such as intra-airway relative humidity and temperature.

3. Single anatomy, truncated at G3 — One subject (CT-based) with Weibel-engineered distal branches; include ≥2 additional anatomies and/or extend generations, or state generalizability limits.
– I agree with the reviewer. This is an important limitation and was partially discussed (in the original version of the manuscript) in the penultimate paragraph under Discussion. Following the reviewer's comments, I have re-worded that paragraph (see lines 684 – 687 and line 691, on pages 22 – 23 in the marked copy). The reviewer's critique has helped clarify the message around the possible lack of generalizability.

4. Rigid walls / no FSI or mucosal motion — Add a compliant-wall (or FSI) sensitivity to capture deformation effects; otherwise declare as a modeling limitation.
– I agree. The lack of dynamic wall effects in the model is an important limitation and was discussed in the 1st revision. However, the reviewer's comment has helped clarify the message further. Please see lines 605 – 607 and lines 617 – 619, on page 20 of the marked copy. Also, see lines 605 – 617 from the 1st round of revision.

5. Single breathing condition (15 L/min, quiet inspiration) — Report sensitivity to flow rate and breathing waveforms (deeper breaths, tachypnea, pauses). If not feasible, mark as a limitation.
– I appreciate the critique. The specificity of the solved breathing rate is indeed a limitation. I have now expanded on it explicitly in a new paragraph under Discussion (see lines 650 – 657, on page 21 of the marked copy).

6. Simplified particle source model — Particles are seeded uniformly at inlet facet centroids; replace with physiologic source maps and realistic size spectra, or state as a limitation.
– I thank the reviewer for this comment. I have added a new paragraph under Discussion, to go over the constraints around the simplified particle source scheme. Please see lines 658 – 664, on page 21 of the marked copy.

7. DPM boundary-condition assumptions — Using reflect (inlet), escape (outlets), trap (walls) can bias trajectories; add a boundary-condition sensitivity check. If not possible, list as a limitation.
– This is also an extremely valid critique. Accordingly, I have added lines 664 - 672 (on page 21 of the marked copy) to expand on the limitations around the assumed DPM boundary conditions and on related future studies.

8. Reduced-order 2D model (S-ROAM) calibration — The point-vortex 2D idealization needs clearer calibration vs 3D LES (errors/uncertainty bands); otherwise state its scope as limited/illustrative.
– I thank the reviewer for the inputs on the novel S-ROAM analysis. While the idealized 2D reduction and the full-scale 3D LES data match qualitatively, I agree with the reviewer on the calibration differences. I have described this limitation in a new paragraph under Discussion (see lines 673 – 683, on page 21 in the marked copy).

9. Viral-load projection assumptions — Fixed sputum concentration, "one particle per breath per size," and T=3 days are strong assumptions; add parameter sweeps (V, generation rate, δt, T). If not feasible, declare as a limitation.
– These are some very important points and hint at the described framework's future scope. To respond, I have drafted a new subsection under Discussion (see lines 572 – 592, on pages 19 – 20 in the marked copy), titled "On the translational inputs for evaluating URT-to-LRT pathogen transport", covering all the limiting assumptions mentioned by the reviewer.

10. Scope ends at G3 (no alveolar deposition) — Clarify that alveolar kinetics are out of scope; if not extending,

state this explicitly as a limitation affecting translational conclusions.
– Thanks for this comment. I have added an explicit mention of this limitation (that the geometry includes till G3 bronchial generation with the alveolar kinetics out of scope, which can affect translational conclusions) in lines 633 – 638 (page 21 in the marked copy), under Discussion. Lines 51 – 53 in the Introduction (unchanged from the original version) also mention the study's limitation related to alveolar deposition analysis.

Additional note: Summary of all the preceding points have been further included in the new Table 3 that provides a domain-specific, itemized list of potential directions for future research that can help resolve the current limitations and enhance the described URT-to-LRT transmission framework. See page 22 of the marked copy.

# Reviewer 2: Minor Revision

The author provided a detailed explanation of my comments; however, I have one concern regarding the discussion of velocity and particle boundary conditions within the geometry. The author did not adequately address this in the revised manuscript. Specifically, it is essential to specify the type of boundary conditions at the inlet, such as a parabolic velocity profile for the velocity and a uniform particle distribution for the particles, as well as to provide similar details for the outlet. Merely naming the reflect and escape boundaries in a numerical paper is insufficient and does not meet the expected standards of clarity and rigor. The paper can be accepted contingent upon this minor revision being made.
– I thank the reviewer for the positive comments and for supporting this manuscript. The starting distribution of the particulates at the laryngeal inlet followed the mesh facet distribution therein. Also the velocity profile was not constrained to be parabolic; rather it was the natural outcome of the governing fluid dynamics equations with the enforced boundary conditions. For the new text detailing the requested inlet/outlet conditions, please see: (a) lines 199 – 202 on page 7 of the marked copy; and (b) lines 285 – 292 on pages 8 – 9 in the marked copy.

# Other notable modifications in the 2nd round of revision

– The functional representation was slightly tweaked (without any change in mathematical implication) in Equation 18, to avoid confusion between η_d (i.e., the transmission efficiency beyond G3 to the deep lungs) and $\eta(d)$.

– New citations: #12, #28, #54, #75, #77, #78, #79 (highlighted with green in marked copy's reference list)

– Additional text in the Introduction (lines 34 – 36, see marked copy) to enhance clarity

– The color coordination in the tables has been slightly tweaked for visual consistency.

Changes in the 1st round of revision (prior revision)

Note: The page numbers in the following description have been updated from those in the Round 1 rebuttal letter to align with the revised manuscript from Round 2.

Point-by-point responses: Reviewers' / Editor's comments (in black) + Author's responses (in blue)

# Reviewer 1

General Summary: Saikat presented The full-scale numerical transmission trends are consistent with findings from our reduced-order mathematical model that conceptualizes the influence of intra-airway vortex instabilities

on local particle transport through point vortex idealization in an anatomy-guided two-dimensional potential flow domain. The results collectively demonstrate markedly elevated lower airway penetration by URT-derived particulates, even by those as large as 10 and 15 μm. The high viral load, often exceeding the pathogen-specific infectious dose, carried by such droplets into the bronchial spaces of the sample airway provides a plausible mechanistic explanation for the accelerated seeding of secondary lung infection.

Major Points:

1. Abstract: The author mentions the existence of viscoelastic behavior but does not mention how to model it in the manuscript. Please see the following article: https://doi.org/10.1177/02676591221128141

– Thank you for pointing to the work on modeling the viscoelastic behavior of airway mucus. Since the abstract is intended to be without citations (as per PLOS One instructions), I have incorporated the suggested reference into the Introduction and provided the relevant context. Please see lines 7–8 (on page 2) in the marked-up version.

2. Line 144: In equation 4, there is no need to write the density term, given that the flow is incompressible.

– I agree and equation 4 has been revised accordingly. Thank you for noting the redundancy of the density term.

3. Due to the lack of a Nomenclature, it is almost impossible to check the equations listed exactly.

– I appreciate the concern. Accordingly, a new nomenclature list has been added for a select set of key mathematical symbols used in the manuscript. Please see page 13 of the marked-up version.

4. Line 171: Have you obtained the time steps of 0.0002 s based on the Courant number?

– The time-step size was preliminarily guided by prior literature (Ghahramani et al. *Computers & Fluids*, 2017), with its order-wise comparability to the Kolmogorov time scale (relevant for the smallest, most dissipative eddies in a turbulent flow). As noted by the reviewer, the choice could also be argued on the basis of the Courant number (CFL). In the revision: to confirm the feasibility of the applied time-step, $CFL = V_o \, \Delta\tilde{t}/s$ was assessed. Here V_o, being the average air velocity through the bronchial outlets, is estimated as Q/A_o, where Q is the target volumetric flow rate of 15 L/min and $A_o = 148.65 \times 10^{-6}$ m^2 is the total open cross-sectional area at the outlets (the latter is extracted from the digitized geometry). Next, $s = 4.73 \times 10^{-4}$ m is the mean spatial grid size in the tetrahedral mesh modeling the bulk cavity. It is calculated as $(6\sqrt{2}\,\Omega)^{1/3}$ (using the volume formulation for a tetrahedron), where Ω represents the mean cell volume calculated by dividing the total airspace volume by the net number of tetrahedral elements in the built mesh. Finally, $\Delta\tilde{t}$ is the implemented time-step size. Using these parameters in consistent units, the estimated CFL is approximately 0.71. The condition $CFL < 1$ indicates that the flow travels less than one grid cell per time-step, which is favorable to numerical stability, particularly in explicit integration schemes, and suggests that the chosen time-step is appropriate for reliably capturing the flow dynamics.

The above details are now added to the Methods section; see lines 204 – 224 (on page 7 of the marked-up copy).

5. Line 211: Have you considered 1622 particulates based on the number of inlet compute nodes?

– Yes, 1622 particulates of each test size were tracked; the count is based on the number of mesh facets (faces) that made up the inlet surface in the redacted geometry. The starting locations of the particulates were at the centroids of the facets. For clarity, panel (l) is added to Figure 2 to show a magnified top view of the inlet surface, demonstrating 400 quadrilateral facets and 1222 triangular facets. Figure 2's caption has been updated accordingly (on page 5). Also, please see lines 282 – 285 (on page 8, in the marked-up copy) for clarifications added to the text.

6. Since you did not consider the deformation of the wall, please mention this as a computational limitation in the discussion. Does this limitation not affect the response? Please see the following article: https://doi.org/10.1016/B978-0-12-823913-1.00009-9

– I thank the reviewer for the suggestion to discuss on the computational limitation arising from the structural

rigidity of the airway enclosure. Wall deformations (including airway compliance, cyclic expansion / contraction, bronchoconstriction or collapse / re-opening) can alter local flow patterns, velocities, residence times, and near-wall mixing, which in turn may impact particulate transport and deposition trends. For instance, rapid area or direction changes would increase inertial impaction (especially important for particulates $\gtrsim 5\ \mu$m), longer residence or flow recirculation can enhance gravitational settling for smaller sizes, and unsteady motion can boost convective–diffusive capture of sub-micron particulates. The net effect depends on the particulate size, airway shapes, and the specific nature and timing of the deformation. In general, this study implicitly assumes that the time scales for such deformations are longer than the time scale of aerial particulate transport within the redacted geometry.

The revision includes a new separate paragraph on the above computational limitation arising from the assumed structural rigidity; please see lines 605 – 617 (on page 20 of the marked-up version) in the Discussion section. The suggested reference has also been incorporated into the new paragraph.

7. Add more details about mesh quality, boundary layer height, Y-plus quantity, etc.
– I appreciate the suggestion from the reviewer to add more details on the mesh parameters. This will help improve the quality of this manuscript. In our test domain, the flow simulations yielded the following results: (a) a mean wall y+ of 0.796 when considering the entire airspace boundary (excluding the inlet and outlets); and (b) a mean wall y+ of 0.825 when focusing only on the bronchial region. These values suggest that the mesh near the wall is well within the typical range for boundary layer resolution, as the target is usually $y+ < 1$ for accurate near-wall modeling. This adequate boundary layer resolution supports the reliability of the numerical results. In addition, the ICEM mesh quality (for the grid selected from the sensitivity analysis) was evaluated. Only 0.283% of the total elements fell into the lowest quality bin, which ranges from 0.35 to 0.38. The low percentage in this bin, combined with higher quality scores for the majority of the mesh elements and the stable convergence trends in the flow simulation (reported in lines 228 – 229), indicates that the overall mesh quality is acceptable.

The above, along with related definitions, has been incorporated as a new separate subsection in the Methods section; see lines 234 – 251 (on pages 7 and 8 of the marked-up copy).

8. In Figure 6, are the Vorticity changes up to 3000 reasonable on the border?! Please explain.
– I appreciate the question. Such high vorticity regions (with magnitudes reaching $\approx 3000\ \text{s}^{-1}$) are observed along the borders. This localized fluid swirling can arise in intricate airway regions, impacting transport and mixing. In the present system, the local rotation of inhaled air is driven by the separating shear layers as the shape of the airway cavity widens following the laryngotracheal constriction.

The revision includes the above explanation; please see lines 499 – 503 (on page 17) of the marked-up copy.

9. Please present the ethics approval document and explain how much radiation dose and in how many layers you were able to obtain the lower airway generations.
– I appreciate these very important questions and requests from the reviewer. This study has used existing, de-identified, high-resolution, medical-grade CT imaging, with slices acquired at coronal depth increments of ≈ 0.4 mm. The CT scanning had exposed the subject to an additional radiation dose of 84 mrem, which is roughly equivalent to the natural background radiation that most individuals receive over four months. Please note that the retrospective use of the anonymized data (resourced from the Department of Otolaryngology/Head and Neck Surgery in the School of Medicine at the University of North Carolina Chapel Hill) for computational analysis was approved under an exempt status by the Institutional Review Board (IRB) at South Dakota State University. The corresponding IRB determination number is: IRB-2206003-EXM.

The above details have been included in the revision (including a cited download link for the IRB documentation); please see lines 95 – 104 (on page 4) in the marked-up version, in the Methods section.

10. Line 167: Specify the numerical value of the pressure inlet and outlet boundary condition.
– Thanks for the suggestion. The pressure conditions have been added in the current lines 198 – 199 and 202 – 204 (on page 7, in the marked-up copy). More details have been added on lines 410 – 413 (on pages 14, 15 in the marked-up copy).

Reviewer 2

Summary: This study investigates the role of microdroplets derived from mucosal liquids in the upper respiratory tract and their contribution to the rapid onset of deep lung infections following upper respiratory tract (URT) infections. Utilizing three-dimensional airway reconstructions and airflow simulations. While this is a robust study with well-discussed results, several issues must be addressed prior to publication in PLOS ONE:

1. It appears that the primary, secondary, and tertiary bronchi were not extracted from CT scan images, which may influence the particle deposition fraction in these regions.
– I thank the reviewer for this comment and for the opportunity to add the relevant details in the revision. The segmentation of the DICOM (Digital Imaging and Communications in Medicine) format CT scans was performed on the image processing software Mimics Research 18.0 (Materialise, Plymouth, Michigan). While the scanned data guided the shapes of the primary bronchi (with the right main bronchus bearing a wider diameter than the left), the subsequent generations of secondary and tertiary bronchi were digitally engineered into the test domain following Weibel's model of repeated bifurcations of the human respiratory tree. The final geometry was quality-checked by a respiratory care specialist at the author's institution (see Acknowledgments) for anatomical realism.

The above details have been added on lines 107 – 115 (see page 4 of the marked-up version), in the Methods section.

Next, in context to the impact of the geometric simplification to the particulate deposition fractions: the smooth surface design of the secondary and tertiary bronchi may (indeed) be construed as a limitation to the overall physiological realism of the model. However, it is important to recognize that the central finding of this study is that larger respiratory particulates, fractured from the upper airway mucosa, can effectively reach the lower airspaces—demonstrating greater penetration compared to particulates of similar sizes inhaled from external sources. Although surface topology details (such as those in the secondary and tertiary bronchi) may influence local deposition sites owing to flow-wall interactions, these nuances are unlikely to significantly affect the overall penetration of particulates into the lower respiratory tract.

The above points are added as a new paragraph in Discussion (lines 620 – 633 on pages 20 – 21, in the marked copy).

2. The findings related to deposition efficiency in the trachea and various bronchi should be compared with previous studies involving inhaled particles to highlight any differences.
– I appreciate the suggestion to compare the reported simulated trends with experimental data. Accordingly, sample published data from figure 5 in https://doi.org/10.1016/j.medengphy.2017.03.004 (Miguel, *Medical Engineering & Physics*, 2017) was used for this comparison. The simulated and experimental trends for bronchial penetration (η %) showed good agreement when plotted on the Stk/Re domain. In addition, considering a deviation of $|\eta| = 2\%$ from the experimental domain as indicative of disagreement, 80% of the numerical data fell within the experimentally prescribed range. The agreement increased to 100% when the permissible deviation was extended to $|\eta| \to 4\%$. In the revised manuscript, Figure 5g (a new panel added to the earlier version of this figure) graphically reports the results from the experimental comparison.

The above details, together with the relevant mathematical elements, are now added as a new subsection under

Results, in lines 476 – 495 (page 17) in the marked-up version. The conclusion (see lines 703 – 705 in the marked-up version) and the abstract are also slightly updated to reflect the addition.

Minor Comments:

1. In the numerical section, providing more detailed information regarding the generation of grid meshes and the numerical equations solved on these grids would be beneficial. Additionally, a more comprehensive explanation of the numerical methods employed would enhance clarity.
– I appreciate the suggestion from the reviewer to incorporate additional details on the mesh generation and the numerical methods. Expanding on the meshing parameters, the revised manuscript now includes details on: (a) the meshing software used (see lines 123 – 126, on page 4, in the marked-up copy); (b) the assessment of mesh quality (see lines 234 – 251, on pages 7 and 8, in the marked-up copy).

Next, expanding on the numerical techniques, the revision includes: (a) an analysis of the chosen time-step size based on Courant number (see lines 204 – 224, on page 7 in the marked-up copy); (b) correction to equation 4 (see lines 169 – 170 in the marked-up copy); (c) additional notes on the Kolmogorov length / time scales and the grid scales (see lines 154 – 155, 205 – 208); (d) pointing out the software package used for the simulations (see line 193 in the marked-up copy); (e) pressure values at the inlet and outlets (see lines 198 – 199 and 202 – 204 in the marked-up copy); (f) nomenclature list for select mathematical symbols (see page 13 in the marked-up copy).

2. The velocity and particle boundary conditions need to be specified for various regions to ensure accuracy.
– I thank the reviewer for the recommendation to clarify the velocity and particle boundary conditions. The airway enclosing walls are assumed to be stationary with no-slip condition. Therein, particulate deposition was tracked through implementation of 'trap' discrete phase model boundary condition. In addition, the discrete phase boundary condition type was set as 'reflect' for the inlet in the redacted geometry and 'escape' at the outlets.

The above details have been added to the Methods section; see lines 274 – 279, on page 8, in the marked-up copy.

3. In Figures 3 and 5, the y-axis of the contour plots appears to be of limited utility. It may be more effective to present this data in an x-y plot, with the x-axis representing diameter and the y-axis representing η or η_d, as this would better illustrate the trends, similar to Figure 5c. Furthermore, it would be preferable to report the results for each generation separately.
– This is a great suggestion from the reviewer. Accordingly, I have converted both Figures 3 and 5 to the x-y format; with the transmission percentages plotted along the y-axis and the particulate diameters along the x-axis. Furthermore, Figure 5 (reporting the main findings on upper airway – to – lower airway transport) has been segregated to separately report: η_p (deposition percentages along the primary bronchi), η_s (deposition percentages along the secondary bronchi), η_t (deposition percentages along the tertiary bronchi), and η_d (penetration percentages beyond the bronchiolar outlets to the deep lungs). The separate zones for the primary and secondary bronchi are shown in panel (c) of revised Figure 1. Please note that η_p has been redefined to exclusively account for primary bronchi data, while η_s is a new symbol separately quantifying the secondary bronchi data. The captions and call-outs to Figures 3 and 5 have also been updated to reflect the changes.

4. The results presented in Table 1 should also be delineated for each generation individually.
– I thank the reviewer for this suggestion. Table 1 (on page 13 in the marked-up version) has been accordingly revised to report the data from each generation separately. In the updated content, n_p gives the count of particulates of each size depositing at the primary bronchi, n_s gives the count of particulates depositing at the secondary bronchi. Previously, n_p was cumulatively quantifying both primary and secondary bronchi data. New particle tracking was done to confirm the distribution of deposits between the primary and secondary bronchi.

Concluding comment from Reviewer 2: Addressing these revisions would significantly enhance the clarity and impact of the study.
– Thank you for your helpful comments. I hope the revised elements will enhance the overall quality of this paper.

Additional comments from the reviewers

1. Is the manuscript technically sound, and do the data support the conclusions?
Reviewer 1: Yes   Reviewer 2: Yes

2. Has the statistical analysis been performed appropriately and rigorously?
Reviewer 1: Yes   Reviewer 2: Yes

3. Have the authors made all data underlying the findings in their manuscript fully available?
Reviewer 1: No   Reviewer 2: Yes
– Author's response: All data are (now) fully available without restriction, through the online open access repository **figshare**; see https://doi.org/10.6084/m9.figshare.29954564. The IRB documentation is also uploaded there.

4. Is the manuscript presented in an intelligible fashion and written in standard English?
Reviewer 1: Yes   Reviewer 2: Yes

Miscellaneous: Other notable modifications by the author

– Figure 5f graphically plots the viral load transmitted, L_v. This visual provides a more comprehensive description of the virion transmission levels, beyond the discrete L_v values reported in Table 2.

– Additional clarifying comments in the Conclusion: (a) on the restricted motion of particulates that are inhaled from outside, see lines 717 – 718 in the marked-up copy; (b) on possible corrections to the potent viral load derivation if we are to account for immune responses, see lines 754 – 755.

– All new references are highlighted in blue background in the marked-up copy. In total, there are 7 new citations (in round 1).

Editor's suggestions / Journal requirements

1. Please ensure that your manuscript meets PLOS ONE's style requirements, including those for file naming.
– I have revised the format of the author affiliation details to adhere to the journal requirements.

2. Please note that funding information should not appear in any section or other areas of your manuscript. We will only publish funding information present in the Funding Statement section of the online submission form. Please remove any funding-related text from the manuscript.
– I have removed the statement on funding from the main manuscript.

3. Thank you for stating the following financial disclosure: "National Science Foundation CAREER Award (Grant Number 2339001; Fluid Dynamics program with Dr. Ron Joslin as program manager)" Please state what role the funders took in the study. If the funders had no role, please state: "The funders had no role in study design, data collection and analysis, decision to publish, or preparation of the manuscript." If this statement is not correct you must amend it as needed. Please include this amended Role of Funder statement in your cover letter; we will change the online submission form on your behalf.
– I have updated the funding statement in the cover letter (enclosed), with the recommended wording.

4. Thank you for uploading your study's underlying data set. Unfortunately, the repository you have noted in your Data Availability statement does not qualify as an acceptable data repository according to PLOS's standards. At this time, please upload the minimal data set necessary to replicate your study's findings to a stable, public repository (such as figshare or Dryad) and provide us with the relevant URLs, DOIs, or accession numbers that may be used to access these data. For a list of recommended repositories and additional information on PLOS standards for data deposition, please see https://journals.plos.org/plosone/s/recommended-repositories.
– Thanks. All data are (now) fully available without restriction, through the online open access repository **figshare**; see https://doi.org/10.6084/m9.figshare.29954564, with doi: 10.6084/m9.figshare.29954564.

Concluding remarks from the author:
I again thank the reviewers and the Editor for their constructive suggestions. I hope that the revised version meets your approval for publication as a research article in PLOS One.

Sincerely,

Saikat Basu, Ph.D.
Associate Professor of Mechanical Engineering
Director, Biomedical & Bioinspired Fluid Dynamics Lab
South Dakota State University
Group Webpage | Faculty Profile | Google Scholar
